# Cellulose Nanofibers Derived Surface Coating in Enhancing the Dye Removal with Cellulosic Ultrafiltration Membrane

**DOI:** 10.3390/membranes12111082

**Published:** 2022-10-31

**Authors:** Luis A. Soto-Salcido, Ikenna Anugwom, Mika Mänttäri, Mari Kallioinen-Mänttäri

**Affiliations:** Department of Separation Science, LUT School of Engineering Science, LUT University, P.O. Box 20, 53851 Lappeenranta, Finland

**Keywords:** surface coating, cellulose nanofibers, methylene blue, ultrafiltration membranes, dye removal

## Abstract

Commercially available ultrafiltration membranes were coated with cellulose nanofibers (CNFs) produced from softwood pulp by a two-step process: a non-derivatizing DES treatment and a simple mechanical treatment (high-speed homogenization and sonification). The CNFs coating aimed at enhancement of the removal of methylene blue (MB) from water and was investigated at different concentrations of the coating, quantified in grams of CNFs per square meter of the membrane (1.3, 6.5, 13, and 19.5 g/m^2^). The pure water permeability (PWP) was unaffected up to the concentration of 6.5 g/m^2^ but the dye retention increased approximately 2.5-fold. Even higher improvement of MB removal, about 4-fold, was observed when 19.5 g/m^2^ were used, however, the pure water permeability also decreased by about 30%. In addition, it was proved that the coating can be removed and created again several times which shows that the concept could be used to improve the retention of organic compounds when high permeability membranes are used.

## 1. Introduction

Dye-containing wastewater from textile, pharmaceutical, paper, and food industries can lead to serious ecological and public health problems due to the coloration of the discharged effluents and the chemical composition of most dyes [1]. The annual estimation of soluble dye production reaches up to 700,000 tons [2]. Methylene blue (MB), 3.7-bis(dimethylamino)phenothiazinylium chloride, is the most common phenothiazinium dye. It is commonly used in the textile industry to dye cotton and silk, and it is often used to represent typical cationic dyes in the literature [3,4]. Direct discharge of this dye into water streams is not recommended because of its toxicity and low biodegradability [5]. Studies have shown that dyes can be removed from effluents by several methods including photocatalytic degradation [6], electrocoagulation [7], adsorption [5,8] flocculation-coagulation, oxidation, and recently, membrane technology [9]. The current methods used in dye removal have several disadvantages. For instance, the photocatalytic degradation method suffers from low dye removal efficiency due to the complex molecular structure of the dyes and the generation of toxic by-products. Furthermore, the high disposal cost of the generated sludge poses a big challenge for the flocculation-coagulation method [10,11]. Pressure-driven membrane filtration processes have emerged as a promising method for dye separation, because in the membrane filtration process, no toxic by-products or sludge are produced as in other dye removal techniques [10]. The efficiency of membranes to remove organic pollutants often depends on the pore size of membrane and size of the organic molecule [12,13]. In addition, electrostatic repulsion might significantly affect the separation when a membrane and molecule have the same sign of charge [13]. For instance, nanofiltration membranes can retain up to 95% of dyes from wastewater, but the operating conditions can result in an expensive process [13]. UF would be a more attractive option for the dye removal if it could retain dye molecules better because it could produce higher volumes of purified waters at lower operational pressure that results in a lower cost process. However, molecular weight cut-off (MWCO) values of UF membranes are larger than the molecular size of the dyes, resulting in poor dye retention [2,10]. To reach the high retention of dye molecules with reasonable filtration capacity and low operational pressures, hybrid approaches have been employed to improve the retention of dyes using UF membranes. In a study by Fradj et al. [9], the MB retention of a regenerated cellulose UF membrane was enhanced from 13 to 98% by mixing two complexing agents (anionic polyelectrolytes) with the dye solution during the filtration in a batch mode cell. In a more recent study, the authors used cross-linked dicarboxymethyl cellulose for MB removal [4]. One of their approaches was to add the cellulose-based adsorbent to the dye mixture during a filtration process using commercial UF membranes; nevertheless, the removal increased only from 90 to 95%.

Recently, various researchers have investigated the use of coatings that can be removed from the surface of membranes [14,15,16,17]. For instance, fouling in reverse osmosis membranes was prevented by using a coating prepared by LbL deposition of polyelectrolytes. Once the membrane was fouled, both the polyelectrolytes coating and fouling film were removed by a simple washing with an alkaline sodium chloride solution [16]. This kind of coating layer, which has the ability to adsorb organic molecules such as dye molecules, might be an interesting option also to improve the retention in an ultrafiltration process. Over the past decade, special attention has been given to nanocellulose-based materials on the surface modification of membranes [18,19]. Cellulose nanofibers (CNFs) are cellulose fibrils with an average diameter less than 100 nm and a length of up to several microns [20,21]. They are versatile biomaterials that exhibit remarkable properties including mechanical and thermal stability, biodegradability, low toxicity, and high surface area [20,22]. They could be utilized on the UF membrane surface as a secondary, removable layer.

CNFs can be produced by mechanical, chemical, and enzymatic methods [23]. The main drawbacks rely on the energy consumed during the mechanical treatment, which elevates the production cost, and the generation of toxic by-products by chemical approaches [24,25]. In this sense, a novel type of solvents known as deep eutectic solvents (DES) provide a greener aid to production of nanocellulose. These kinds of solvents result from the eutectic mixture of at least one hydrogen-bonding acceptor (i.e., a quaternary ammonium salt) and at least one hydrogen-bonding donor (i.e., carboxylic acid, alcohol, or amine). DES have been recently used to prepare nanocellulose by a non-derivatizing treatment from different lignocellulosic resources [26,27,28,29]. For instance, Sirviö et al. [26] used the eutectic mixture of choline chloride and urea to pretreat cellulose pulp before using a microfluidizer. A recent study explored DES pretreatment with choline chloride and lactic acid [29] as an initial step for CNFs production from Moso bamboo, followed by high-speed homogenization and microfluidization. As result, widths ranging from 20 to 80 nm were observed. In the present work, we proposed a similar DES-pretreatment (choline chloride: lactic acid), and high-speed homogenization, however, ultrasonication was used instead of a mechanical disintegration with a microfluidizer.

CNFs have been shown to function as adsorbents for dyes [8]. Additionally, they have been applied as nanomaterials in biomedicine [30] as well as in the production of nanocomposites for food packaging [31]. Furthermore, they have been applied in membrane technology [18,19]. Gold nanoparticles and ferritin were rejected over 90% when microfiltration (MF) filter papers were coated with chemically prepared CNFs. Moreover, MB was proved to be adsorbed by the CNFs at 80.57 mg/g of capacity [18]. In a more recent study, UF-like membranes were prepared by coating MF membranes with TEMPO-oxidized CNFs and polyvinyl alcohol. As a result, the modified membranes exhibited 75% less adsorption of bovine serum albumin (BSA) [19]. However, to date, the use of CNFs as removable adsorptive coating in UF membrane systems has not been investigated.

The purpose of the study was to demonstrate that the CNFs can be prepared through an environmentally friendly DES-aided defibrillation treatment followed by a mechanical treatment and the prepared CNFs can be utilized as a replaceable coating in commercial cellulose regenerated UF membranes to enhance the removal of micropollutants’ dye molecules without sacrificing permeability of the membrane.

## 2. Materials and Methods

### 2.1. Materials

The cellulose source was spruce wood pulp pretreated by DES [32]. The DES used for the non-derivatizing pretreatment was composed of choline chloride (99%, Acros organics, Geel, AN, Belgium) and lactic acid (90%, VWR Chemicals, Radnor, PA, USA) with the molar ratio of 1:9. The surface coating experiments were performed on the commercial regenerated cellulose UF membrane RC70PP, which is manufactured by Alfa Laval. A 10 ppm methylene blue (MB) (J.T. Baker, Holland) and 10 ppm reactive orange 16 (Sigma-Aldrich, Darmstadt, Germany) solutions were used in the experiments to test the dye removal efficiency.

### 2.2. Preparation of Cellulose Nanofibers

The spruce pulp was pretreated using a DES composed of choline chloride and lactic acid (1:20 molar ratio) during 5 h at 120 °C in a weight ratio of 1:20 (biomass/DES); the resulting material was washed with abundant water and dried at 55 °C. Samples of 0.1% (*w*/*v*) were hydrated with pure water and stirred overnight. A 400 mL solution was homogenized for 1 h at 24,000 rpm at room temperature, with an IKA Ultra-turrax T-25 and, after that, the ultrasonication was set at 100% amplitude and pulse of 6 s for 1 h [33] using a VibraCell VC100 (100 W, 20 kHz) by Sonics and Materials. To avoid overheating during homogenization and ultrasonication, the solution was placed in an ice bath. The resulting solution was then kept at 5 °C until further use. The dimensions of the CNFs were determined by transmission electron microscopy (TEM) by Hitachi HT7700, sample grid: Cu-grid with carbon coating, the sample was stained with phosphotungstic acid (1 w%), and the acceleration voltage was 100 kV. The surface charge of the CNFs was measured using the Zetasizer ZS Nano Malvern 2013 equipment for a pH range from approximately 9 to 3 using a disposable folded capillary cell.

### 2.3. Cellulose Nanofibers Coating on the Membrane

The amount of CNFs on the surface of the UF membrane was regulated by the volume of a matrix solution used during coating. From a 0.1% (*w*/*v*) solution, four different volumes were used for the coating (5, 25, 50, and 75 mL). The corresponding amount of coating on the membrane surface for each selected volume is shown in Table 1.

#### Coating and Filtration Process

For the coating, the membrane was placed in an Amicon cell (Millipore) dead-end filtration system with 300 mL capacity and an effective filtration area of 40 cm^2^. The membrane was first compacted at 3 bar for 20 min. Then, pure water permeability (PWP) was measured at 1 bar and 22 °C. The PWP was calculated using Equation (1):

(1) $$PWP=\left(\frac{Q}{A\times t\times p}\right)$$

where *A* is the effective filtration area (m^2^), *t* is the sampling time (h), *Q* is the permeate volume (L), and *p* is the pressure (bar). After PWP measurements, the remaining water was removed from the cylindric glass, and the selected volume of the CNFs solution was added. Then, the coating was performed by stirring the solution for 1 min at 150 rpm, and finally, without stirring at 1 bar of pressure, the CNFs solution was filtrated until water had passed through the membrane.

The effect of coating on permeability was analyzed by measuring PWP again after coating. In addition, its influence on MB retention was evaluated. Then, 200 mL of 10 ppm MB solution was poured into the Amicon cell. The filtration process was carried out at 1 bar and at stirring speed of 150 rpm. MB retentions were calculated using Equation (2). MB content in the samples was measured by UV-Vis at absorbance of 664 nm:

(2) $$R\left(\%\right)=\left(1-\frac{2C_{p}}{\left(C_{f}+C_{r}\right)}\right)\times 100$$

where *C_p_* is the total permeate concentration (mg/L), *C_f_* is the total feed concentration (mg/L), *C_r_* is the total retentate concentration (mg/L), and *R* is the retention (%).

Fourier-transform infrared spectroscopy (FTIR) analysis was done for the pristine and coated membranes with a concentration of 6.5 g/m^2^ of CNFs, using a Frontier (Perkin Elmer) FTIR spectrometer equipped with a universal attenuated total reflectance (ATR) module. The resolution was set at 4 cm^−1^ at the absorbance mode of FTIR spectrum. The measurements were performed in the wavenumber range of 4000–400 cm^−1^ with the date interval 1 cm^−1^. The pristine and coated membranes were dried overnight in an oven at 55 °C before the analysis.

### 2.4. Membrane Performance When Coating Was Repeated Several Times

**Figure 1:**
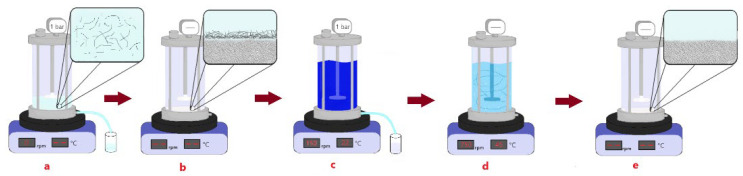
(**a**) Surface coating is performed by filtrating a CNFs solution; (**b**) CNFs are attached to the membrane surface; (**c**) MB retention is tested; (**d**) coating is washed away; (**e**) membrane is free of CNFs and ready for a new coating process.

The replaceability of the coating was investigated using 6.5 g/m^2^. The coating was placed, removed and built up to five times, and the performance in terms of dye removal and permeability was examined each time the CNFs were replaced. In Figure 1, a scheme of the complete process is shown. First, the CNFs solution was placed in the system and was filtrated (a). When all the solution has passed through the membrane, a visible to the naked eye thin film is observed (b), then, the dye is filtrated (c). After MB filtration, the coating layer was easily removed by increasing the stirring from 150 rpm to 750 rpm (d), and finally, the membrane is ready for a new coating (e). The MB retention of a pristine UF membrane was also measured 5 times with solutions of the same concentration.

## 3. Results and Discussions

### 3.1. Preparation of Cellulose Nanofibers

**Figure 2:**
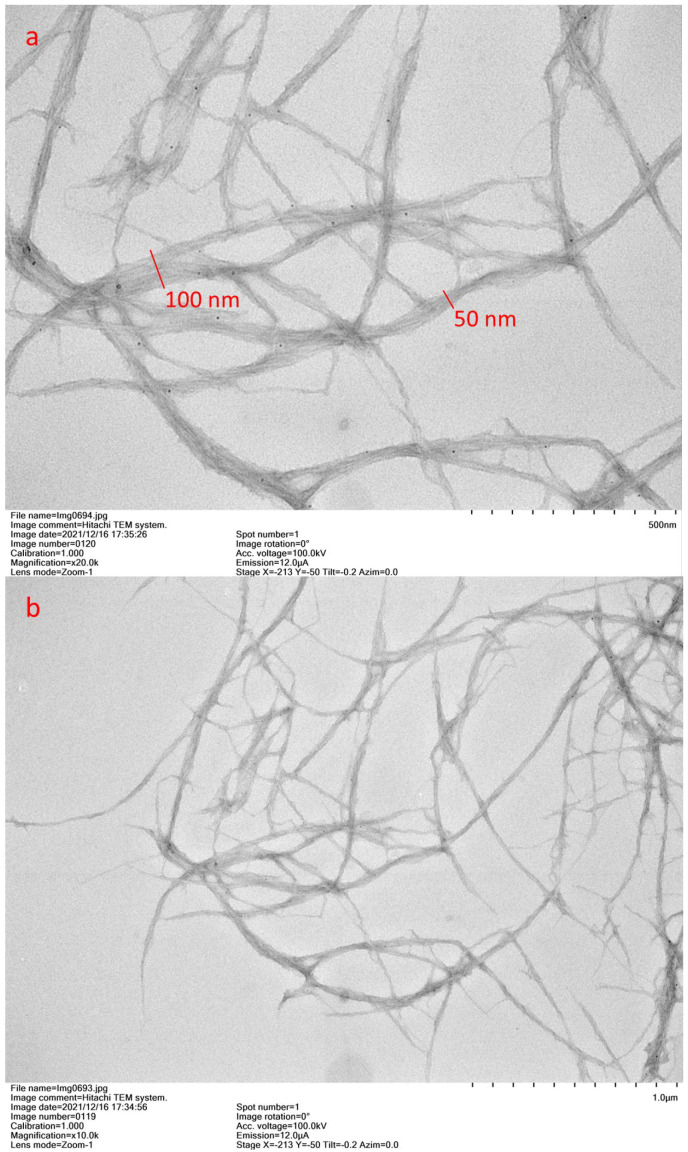
TEM images of prepared cellulose nanofibers: (**a**) 500 nm scale and (**b**) 1000 nm scale.

CNFs were prepared from biomass pretreated with DES (choline chloride: lactic acid). It has been reported that this eutectic mixture enables cellulose fibril to untangle from fibers by weakening the cellulose structure, nevertheless, it is not sufficient for defibrillation in the nanoscale [29]. Thus, there is a need for other treatment steps to achieve the CNFs. Figure 2 shows the TEM images of the CNFs prepared in this study. As can be seen, on average, diameters of the fibers are approximately 100 nm, and their length is in the microscale, in line with the definition of CNFs [20,21]. Fibril diameters from 50 to 100 nm were observed. This result is consistent with the results presented by Liu et al. [29] (20 to 80 nm), who prepared CNFs with a similar DES pretreatment as used here, but with different further steps. Liu et al. [29] did the mechanical disintegration with a microfluidizer, while here, ultrasonication was applied. Recently, ultrasonication, which is considered as a green process [34], has been used for cellulose nanofibrillation. During ultrasonication in a liquid medium, the formation and rapid collapse of microbubbles degrade polysaccharide linkages that promote nanofibrillation of the cellulose [35,36]. Conventional techniques such as high-pressure homogenization and microfluidization require multiple passes and moreover, often suffer from clogging when the fibers are not reduced by a pretreatment (previously defibrillation) [35,36,37]. In this sense, ultrasonication could emerge as an alternative to microfluidization when preparing CNFs. TEM images confirmed that CNFs can be obtained by the novel method proposed, which consisted of a two-step process: a non-derivatizing DES treatment and a simple mechanical treatment (high-speed homogenization and sonification).

### 3.2. Use of CNFs in Membrane Coating

**Figure 3:**
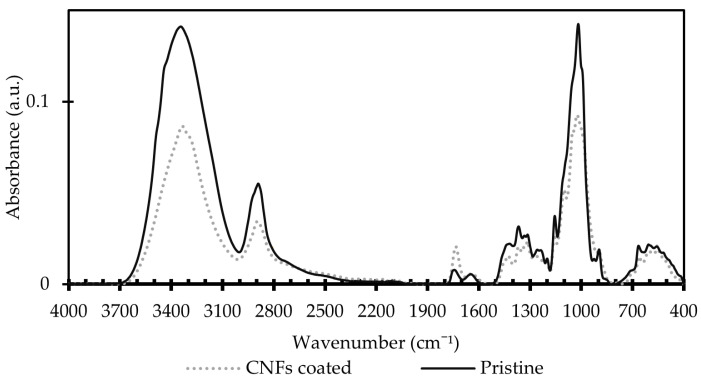
FTIR spectra of the pristine RC70PP and coated with 6.5 g/m^2^ of CNF membranes.

The presence of the coating was visible by the naked eye; however, FTIR analysis of the dried samples were made for the pristine and coated membranes with 6.5 g/m^2^ of CNFs. The FTIR spectra of the pristine and coated membranes (Figure 3) was not expected to drastically differ from each other because they are both cellulose-based materials. A slightly higher intensity of the peak at 1730 cm^−1^ indicates that the CNFs-coated membrane have more carboxyl groups than the pristine regenerated cellulose membrane. This could be explained by the presence of residual lactic acid from DES-based defibrillation process, or by the acetylation of a small fraction of the CNFs [38]. The spectra of the CNFs-coated membrane showed a lower intensity of the–OH groups, commonly expressed in the peaks at 3350 cm^−1^ and 1000 cm^−1^ characteristic of cellulose [38] which would be consistent with the shallowed superior content of carbonyl groups in the CNFs coating.

#### 3.2.1. Effect of CNFs Concentration on Membrane Permeability

**Figure 4:**
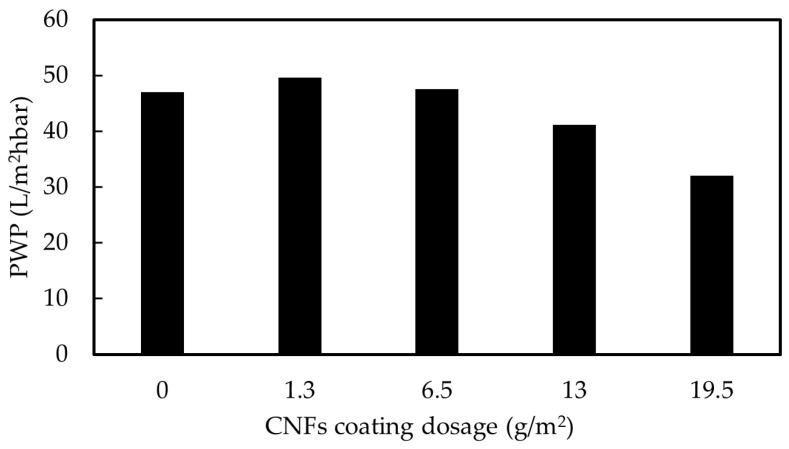
Pure water permeability of pristine and coated UF membranes at different CNFs dosages.

The relation between PWP of the UF membrane and the used coating dosage measured in this study is shown in Figure 4. Initially, native UF membranes presented, on average, a PWP of 46.9 L/m^2^hbar. With the two smallest coating doses used, the PWP was not significantly affected. A minor PWP decrease occurred when a dosage of 13 g/m^2^ was used for coating while the use of a dosage of 19.5 g/m^2^ resulted in a clear decrease in PWP; the PWP displayed was approximately 70% of the one shown by the pristine membrane. The result is reasonable, because the more CNFs there are on the membrane surface, the thicker and denser layer they can form when they are pressed towards the membrane surface during the filtration. The same phenomenon has been reported also by Wang et al. [39]. They coated filter paper with CNF at 60 °C using vacuum drying, and they found that the flux of a filter paper coated with a solution of 0.05% decreased by approximately 18% of its original performance; furthermore, greater flux reductions of nearly 83% and 96% were observed when 0.1% and 0.2% CNF solutions were used, respectively [39].

#### 3.2.2. Effect of CNFs Coating on Methylene Blue Retention

**Figure 5:**
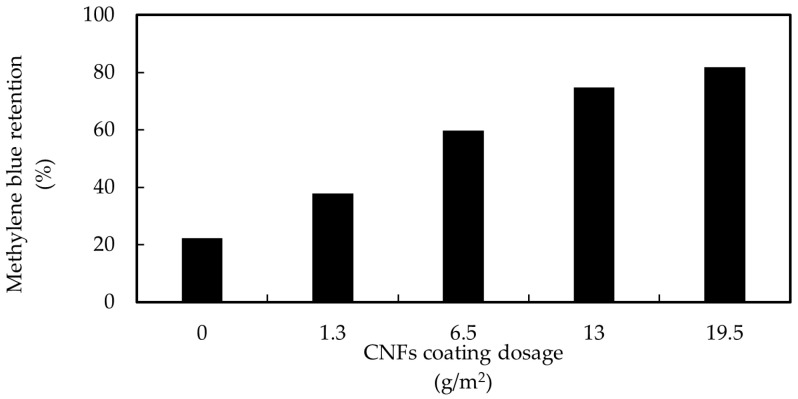
Methylene blue retention of a pristine UF membrane and coated by different CNFs dosages.

A positive correlation was found between the amount of CNFs coating and the MB retention. The more the coating dose was increased, the more MB was retained, as can be seen in Figure 5. The pristine membrane displayed a 22% MB retention. However, when the dosage of 1.3 g/m^2^ was used for the coating, the MB retention increased up to 1.7 times. The highest MB retention, 82%, was shown for the membrane coated with the highest CNF dosage (19.5 g/m^2^). The increased retention could be attributed to the adsorption of the dye onto the fibers. The higher the amount of CNFs used in the coating, the higher was the surface area of the CNFs and the better was the removal of MB. At pH 6.7, the adsorption was promoted by electrostatic attraction of cationic dye and negatively charged CNFs.

**Figure 6:**
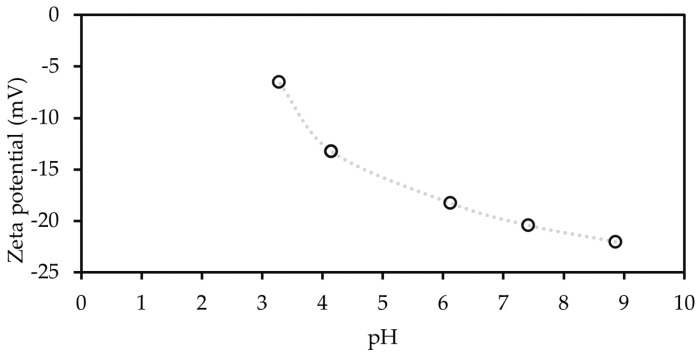
Surface charge of the CNFs particles.

The pH is known to be an important parameter during MB adsorption; Fradj et al. [9] reported that at acidic pH, the removal by UF membranes ranged from 13 to 18% depending on the concentration of MB. Moreover, Chan et al. [24] reported that the removal of MB by adsorption on CNFs increased from approximately 35% to approximately 78% when pH was increased from 3 to 7. The surface charge of the CNFs (Figure 6) expressed by the zeta potential at pH 7 was approximately −20 mV, consistent with other study presented by Kim et al. [40]. The MB is mainly found in its cationic form because of its acid-base equilibrium and low, less than 1, pKa. As a result, at pH 6.7 (condition used in this study), the dye removal was promoted by electrostatic attraction of the unprotonated dye and the negatively charged CNFs [9]. The pristine RC70PP membrane is also negatively charged (zeta potential −27 mV at pH 7) based on the streaming potential measurements reported by Nieminen et al. [41]. This explains the 20% initial dye retention of pristine membrane. However, once the adsorbent sites were full by the MB, the retention was less than 2% (Section 3.2.3).

Therefore, it can be assumed that the dye removal mechanism in this study was mainly due to adsorption on the CNFs coating and a lesser amount was adsorbed on the membrane’s surface. To strengthen the hypothesis, a 10 ppm solution of reactive orange 16, an anionic dye, was separately filtrated by coated membranes with a coating dosage of 6.5 g/m^2^. The coated membrane retained only about 4% of the negatively charged dye. This shows that the pore size of the membrane or coating layer was too large in order to show that the electrostatic repulsion or sieving (molar mass 617.54 g/mol) could have had an effect on the removal of dye molecules. The molar mass of MB (319.85 g/mol) is even lower than reactive orange 16, therefore, it can be concluded that the separation of cationic methylene blue was due to adsorption on the negatively charged cellulose nanofibers.

#### 3.2.3. Replaceability of the Coating

**Figure 7:**
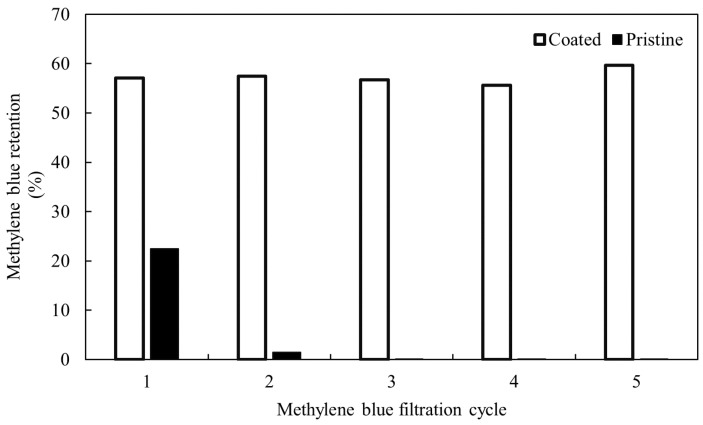
Ten-ppm MB solutions were filtrated by a pristine and coated UF membranes five times. Coating was removed and new coatings of CNFs (6.5 g/m^2^) were built up again after each filtration of fresh dye solution.

The CNFs coating on the UF membrane surface is visible to the naked eye as a crystalline, thin, gel-like layer. The layer is easy to remove by rinsing with water and increasing the turbulence on the membrane’s surface (increase of the mixing up to 1000 rpm) for over 10 min. Moreover, the coating could be removed in only 2 to 5 min by increasing the temperature of water to 45 °C and using a stirring speed of 750 rpm. The flux was recovered after removing the CNFs coating from the surface of the membrane. Along with the flux, MB retention remained similar after five cycles of coating replacement, as can be seen in Figure 7. The initial MB retention for pristine UF membrane was 24% and drastically decreased to approximately 2% during the second cycle, and remained there for the rest of the experiment, meaning that the adsorption capacity of the membrane itself was full after the first cycle, and there was no more significant adsorption on the membrane surface occurring during the rest of the MB filtration tests.

**Figure 8:**
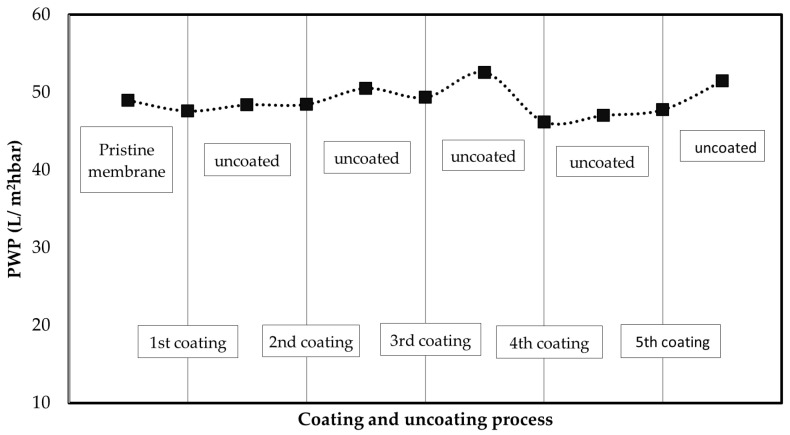
Membrane performance in terms of PWP during coating with 6.5 g/m^2^ of CNFs and coating removal cycle.

The coating of the membrane with 6.5 g/m^2^ of CNFs did not change the PWP of the membrane. In addition, no change in the PWP flux was observed when the coating layer was removed and prepared again. As Figure 8 shows, it is possible to remove the coating layer and reconstruct it again several times. The results prove that CNFs coating can be replaced several times without compromising its performance in terms of water flux or retention.

The replaceability of the coating (6.5 g/m^2^) on the membrane displayed excellent results by maintaining approximately 57% and 48 L/m^2^hbar of dye retention and PWP, respectively. In summary, from the results shown in this study, it can be deduced that CNFs prepared by the method proposed can be used to modify the surface of regenerated cellulose UF membranes. Additionally, the coating can be easily removed and created again on the membrane surface. Moreover, the capacity to enhance the removal of the dye MB in the UF process has been proved. Taken together, the adsorptive properties of CNFs, and the approach used in this study, formulates the hypothesis that coated UF membranes could be utilized to remove certain organic compounds from water without sacrificing the high filtration capacity. However, further experiments are needed to find out how efficiently, for instance, different organic micropollutants including pharmaceuticals, pesticides, and personal care products can be removed with a CNFs-coated UF membrane.

#### 3.2.4. Coating Stability

**Figure 9:**
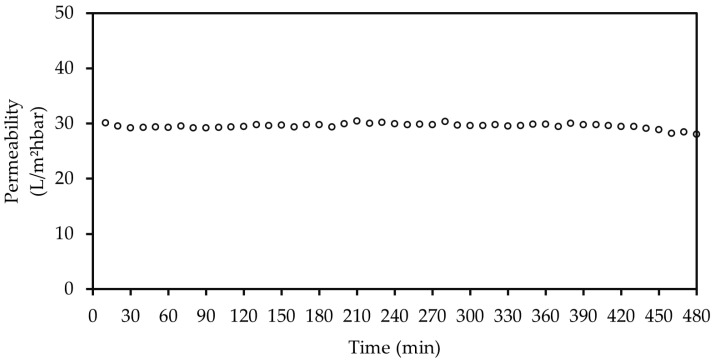
Coating stability of 6.5 g/m^2^ CNFs, at 22 °C and 150 rpm of stirring.

A CNFs content of 6.5 g/m^2^ was used to test the stability of the coating for 8 h as shown in Figure 9. It is noteworthy that a brand new RC70PP membrane was used for this experiment, thus, the permeability of the pristine membrane was approximately 36 L/m^2^hbar, which was lower by the other results presented throughout this study. The permeability remained intact during the whole filtration process, indicating that the CNFs remained stable on the surface of regenerated cellulose membranes for long periods of time. Moreover, the coating layer was visible by the naked eye, which confirmed that the CNFs can last at least 8 h at a filtration condition of 22 °C and 150 rpm of stirring. 

## 4. Conclusions

CNFs were prepared from spruce pulp by a non-derivatizing method using DES, aided by a mechanical treatment, resulting in fibers with an average diameter less than 100 nm, as confirmed by TEM. An easy approach to place the CNFs on and remove them from the surface of commercial UF membranes was developed. The MB retention improved up to 4-fold when the membrane was coated with 19.5 g/m^2^ of CNFs, but the permeability also decreased by 32%. When the lower amount of CNFs (6.5 g/m^2^) was used, the permeability remained intact, but the dye retention increased 2.5-fold. The removal of the coating was performed in over 10 min just by increasing the stirring speed from 150 to 1000 rpm, but the removal could be sped up by increasing the temperature of the water to 45 °C and using a stirring speed of 750 rpm. The replacement and building up of the coating were done up to five times on the same membrane: the MB retention and PWP were similar after each coating. These results suggest that easily removable CNFs coating could be applied on the surface of regenerated cellulose UF membranes to improve the removal of organic compounds such as methylene blue. By coating the UF membrane with CNFs, it was possible to combine the high permeability of UF membrane with the good removal of NF membranes. This enhances the possibility of membrane filtration to respond to water treatment challenges, such as removal of micropollutants, more energy efficiently in future. It can be assumed that, due to the adsorptive removal mechanism, the developed concept is most feasible when the concentration of pollutant is low.

## Figures and Tables

**Table 1:** Volumes of the cellulose nanofiber solutions used in the filtration experiments and the respective amounts of CNFs on membrane surfaces.

CNFs Solution (mL)	Coating Content (g/m^2^)
5	1.3
25	6.5
50	13
75	19.5

## Data Availability

The data presented in this study can be requested from the corresponding author.
